# Supplementary material for: Effect of Shock Waves on the Growth of Aspergillus niger Conidia: Evaluation of Germination and Preliminary Study on Gene Expression
Source: J Fungi (Basel). 2022 Oct 24;8(11):1117. doi: 10.3390/jof8111117 (PMC9699263; doi:10.3390/jof8111117)
Supplement: Supplementary file 1 [file jof-08-01117-s001.zip › jof-1905579-supplementary.pdf]

Supplementary Materials

JOURNAL OF FUNGI

Effect of shock waves on the growth of Aspergillus niger conidia: evaluation of germination and preliminary study on gene expression

Daniel Larrañaga[1], Miguel A. Martínez-Maldonado[2], Blanca E. Millán-Chiu[3], Francisco Fernández[2], Eduardo Castaño-Tostado[4], Miguel Ángel Gómez-Lim[5], Achim M. Loske[2]

[1] Posgrado en Ciencia e Ingeniería de Materiales, Centro de Física Aplicada y Tecnología Avanzada, Universidad Nacional Autónoma de México, Blvd. Juriquilla 3001, 76230, Querétaro, Qro., Mexico

[2] Centro de Física Aplicada y Tecnología Avanzada, Universidad Nacional Autónoma de México, Blvd. Juriquilla 3001, 76230, Querétaro, Qro., Mexico

[3] CONACyT - Centro de Física Aplicada y Tecnología Avanzada, Universidad Nacional Autónoma de México, Blvd. Juriquilla 3001, 76230, Querétaro, Qro., Mexico

[4] Facultad de Química, Universidad Autónoma de Querétaro, C.U., Cerro de las Campanas s/n, 76010, Querétaro, Qro., Mexico

[5] Centro de Investigación y Estudios Avanzados del Instituto Politécnico Nacional, Unidad Irapuato, Km 9.6 Libramiento Norte Carretera Irapuato-León, 36824, Irapuato, Gto., Mexico

Corresponding author:
Blanca E. Millán-Chiu
E-mail address: blanca.millan@fata.unam.mx
Tel.: +52 (442) 192 6126
Fax: +52 (442) 2381165

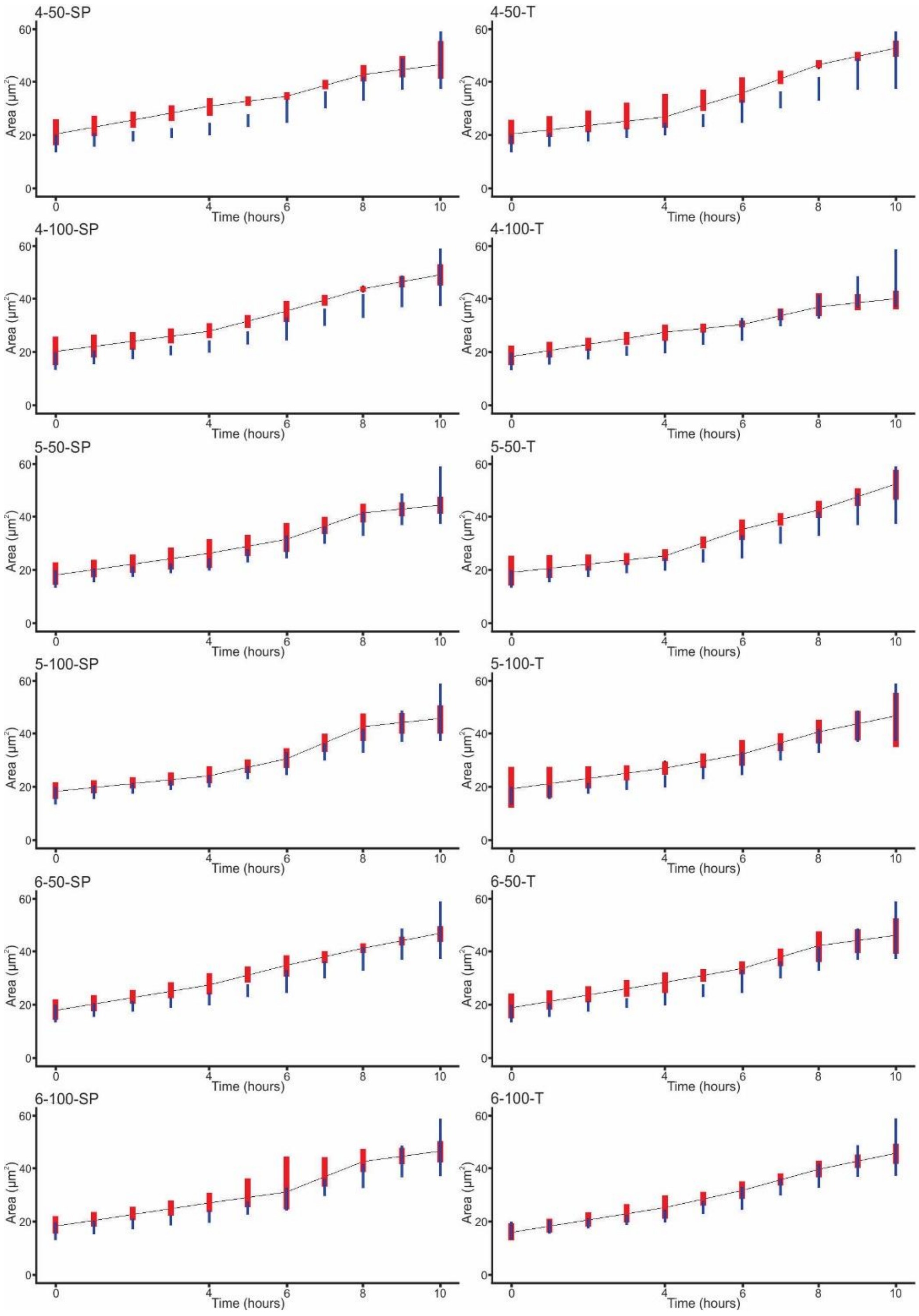

Figure S1. Swelling of conidia versus time after exposure to 50 and 100 single-pulse (SP) shock waves generated at 4 kV, 5 kV, 6 kV, and to 25 and 50 tandem (T) events (50 and 100 shock waves, respectively) generated at 4 kV, 5 kV, 6 kV. Vertical red lines represent 95% bootstrap intervals of the growth of shock wave-treated conidia, and vertical blue lines refer to the 95% bootstrap interval of the control experimental groups. Overlapping intervals indicate a nonsignificant difference.

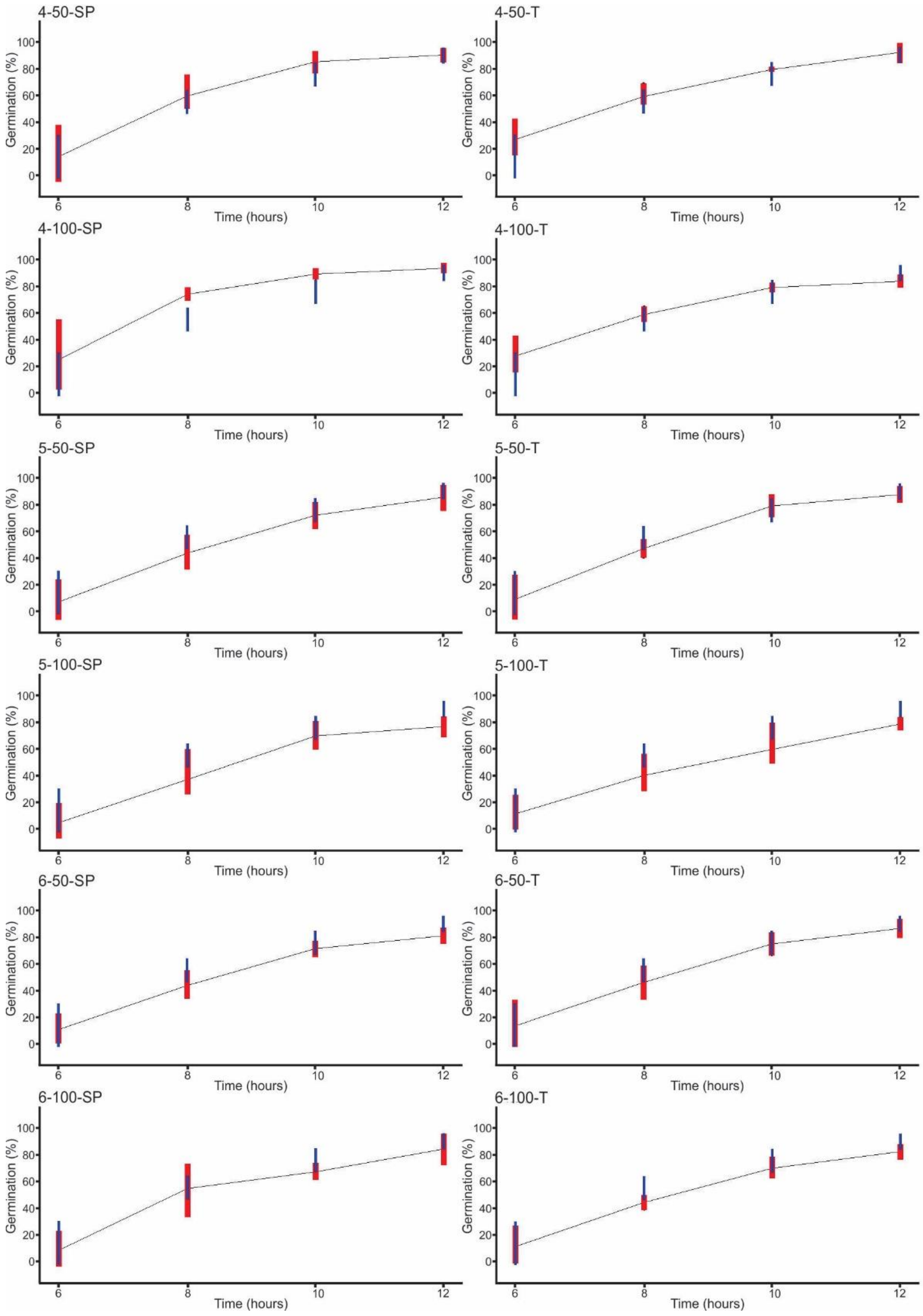

Figure S2. Percentage of conidia that formed a germ tube after being exposed to 50 and 100 single-pulse (SP) shock waves generated at 4 kV, 5 kV, 6 kV, and to 25 and 50 tandem (T) events (50 and 100 shock waves, respectively), generated at 4 kV, 5 kV, 6 kV. Vertical red lines represent 95% bootstrap intervals of the growth of shock wave-treated conidia, and vertical blue lines refer to the 95% bootstrap interval of the control experimental groups. Overlapping intervals indicate a nonsignificant difference.
